# Distribution, source apportionment, and risk analysis of heavy metals in river sediments of the Urmia Lake basin

**DOI:** 10.1038/s41598-022-21752-w

**Published:** 2022-10-19

**Authors:** Salar Rezapour, Farrokh Asadzadeh, Amin Nouri, Habib Khodaverdiloo, Mohammad Heidari

**Affiliations:** 1 Soil Science Department, Urmia University, P.O. Box 165, Urmia, 57134 Islamic Republic of Iran; 2 Hermiston Agricultural Research and Extension Center, Oregon State University, Hermiston, OR 97838 USA; 3 Department of Epidemiology, School of Medicine, Urmia University of Medical Sciences, Urmia, Iran

**Keywords:** Ecology, Ecology, Environmental sciences, Limnology, Solid Earth sciences

## Abstract

The anthropogenic heavy metal dissemination in the natural environment through riverine sediments is a major ecological and public health concern around the world. This study gives insight into the source apportionment and potential ecological and health risks of heavy metals in river sediments of the Urmia Lake basin, a natural world heritage located in northwestern Iran. A comprehensive sediment sampling was conducted in seven major rivers feeding the basin during the summer and winter of 2021. Samples were analyzed for zinc (Zn), copper (Cu), cadmium (Cd), lead (Pb), and nickel (Ni) contents and a suite of chemical and physical properties. Subsequently, Pollution Index (PI), Pollution Load Index (PLI), Ecological Risk (ER), Hazard Quotients (HQ), Hazard Index (HI), and Carcinogenic Risk (CR) indices were determined. The mean concentration of heavy metals in all rivers’ sediments exhibited the descending order of Ni > Zn > Pb > Cu > Cd during both summer and winter. Multivariate analysis suggested that Zn was primarily initiated from natural processes, Cd and Pb were affected by human activities, and Cu along Ni were derived from natural and anthropogenic factors. The PI unveiled that most sediment samples were unpolluted to slightly polluted by Zn, Cu, and Pb, and slightly to moderately polluted by Cd. PLI and ER indices demonstrated that the sediment poses non to moderate pollution and low to moderate ecological risk, respectively. Using a human health risk approach, we found that the HI values of all heavy metals and THI were less than one for children and adults implying non-carcinogenic risk in the analyzed sediments. Carcinogenic effects of Cd and Pb at all rivers sediments via ingestion, inhalation, and dermal contact were almost within tolerable risks (1 × 10^−6^ to 1 × 10^−4^) for children and adults. PI, PLI, ER, HQ, HI, and CR index values of sediment samples during the summer were higher than those during the winter. This is attributed to the greater heavy metal concentrations and the lower water flow during summer. Our results provide practical information for better management and control of heavy metal pollution in aquatic-sedimentary ecosystems.

## Introduction

The accumulation of heavy metals in sediments is a major threat to aquatic ecosystems and other components of the natural environment^1,2^. Sediments are important sinks and carriers of heavy metals in the land–water transition zone, retaining almost 99% of the adsorbed metals after entry into the aquatic ecosystems^3,4^. Circulation of heavy metals in the environment occurs mainly through biogeochemical processes. However, human activities such as mining and manufacturing increase the diversity and concentration of heavy metals in terrestrial and aquatic ecosystems, thereby enhancing the potential of exposure and toxicity for different organisms^5^. Depending upon flow competence, particle size, and basin characteristics (e.g., pH, organic carbon, calcium carbonate, and salinity), flowing water has a substantial role in the dissemination of sediments-associated heavy metals across the water-soil–plant-human domain^6^. Benthic organisms in aquatic environments are more exposed to heavy metals through the bioturbation of sediments. This process re-distributes the heavy metals associated with the underlying sediment, thereby increasing the bioavailability and exposure potential for non-benthic organisms^7^. Migration of fish and transport of metals throughout species within the food chain exacerbates the distribution of sediment-associated heavy metals in aquatic ecosystems^4,8^. Heavy metals attracted by sediments may persist in the natural environment for long periods due to their specific physicochemical characteristics―e.g., high density, non-biodegradability, bioaccumulation, the long half-life, and difficulty to remove via self-purification^4,9,10^.

Heavy metals are naturally initiated by lithogenic processes as well as anthropogenic activities (e.g., atmospheric deposition, wastewater disposal, runoff, industrial wastes, and agricultural effluents)^7,11,12^. Some heavy metals can be dissolved and others may attach to the suspended particles after entering the aquatic environment from various sources, thereby settling in the sedimentary substrate with a progression over time^7,13^. Thus, sediment is a fundamental and dynamic part of the aquatic ecosystems―bearing physical and biogeochemical characteristics that determine the potential environmental risk. The assessment of these traits provides critical information about the significance of heavy metal pollution and guides environmental specialists toward specific remediation strategies^2,14^. Likewise, such information will assist governments, policymakers, and environmental activists to gain insight into the relationships between coastal development and its sustainable management to protect shorelines from worldwide heavy metal pollution^15^.

Heavy metals-enriched sediments may be potential threats to the food chain and human health through different pathways^7^. Oral ingestion, inhalation, and dermal exposure are three major heavy metals entry routes to the human body^16^. Specific metal transporters can move metals from apical to the basolateral surface to circulate throughout the organs by blood. Metals exhibit various oxidation states which facilitate their reactions in biological systems by losing electrons and generating reactive oxygen species (ROS) and free electrons^17^. Metals may be exerted through bile and urine, or they may accumulate in various tissues such as muscles, liver, and kidneys leading to various carcinogenic and other chronic disorders irrespective of age or gender^18^.

Urmia Lake, with a surface area of approximately 5200 km^2^ is the second largest salt lake in the world^19^. This endorheic saltwater lake presents one of the rarest and most diverse biosphere reserves in Iran and the world^20^. The lake has been recognized by UNESCO as an environmental heritage and international wetland^19,20^. Over the past few decades, climate changes have substantially declined the net water discharge into the basin. On the other hand, the rapid growth of industrial units, expansion of residential and agricultural lands, and unsustainable natural resource management strategies have increased the load of heavy metals along with the other contaminants into Urmia Lake through runoff, and sediment transport. It has raised concerns regarding the contamination of shallow aquifers in the basin which are primary freshwater resources for humans, animals, and agricultural water use in the basin. Metals may form a range of more toxic compounds by interacting with nitrates, sulfates, carbonates, and other anions which are found in abundance in agricultural discharge^17,21^. Metals speciation can considerably enhance the bioavailability and bioaccumulation of metallic compounds in organisms, thereby threatening the highly diverse fauna and flora in the region. However, little information is available regarding the heavy metal pollution and associated health risk in the Urmia Lake basin. Improved understanding of the level of heavy metal pollution in sediments provides a critical decision support tool for stakeholders, including both government and the public communities to take proper actions to protect the unique ecological and wildlife ecosystem of the Urmia Lake basin. Therefore, the objectives of this study were to 1) investigate the level, distribution, and accumulation of heavy metals including Zn, Cu, Cd, Pb, and Ni in the river sediments of the Urmia Lake basin during two seasons, 2) assess the contamination rate and human health risks of heavy metals in the sediments of the study region using various evaluation techniques, and 3) explore the potential sources of heavy metals through multivariate statistical analysis.

## Methodology

### Description of the study region

**Figure 1:**
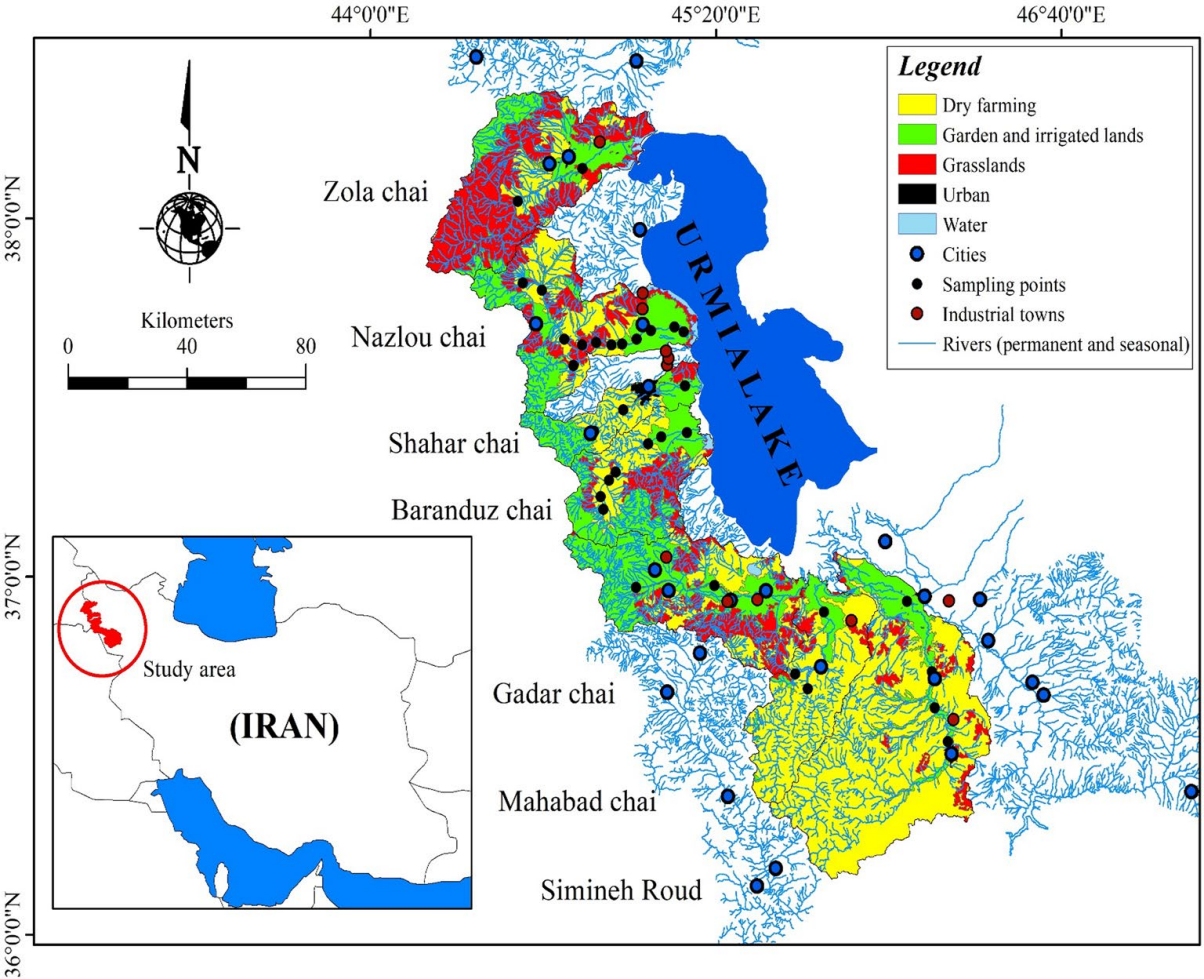
Map of the study region illustrates the rivers feeding the Urmia Lake, sampling points, cities, industrial towns, and prevailing land use practices in West Azerbaijan province, northwestern Iran (ArcGIS Pro, Version 2.5)^74^.

The study region is located in the northwest of Iran (36° 23′ to 38° 15′ N and 44° 32′ to 46° 15′ E) (Fig. 1). Urmia Lake catchment is characterized by semi-arid climate^22^ with a 30-year mean annual rainfall of approximately 364 mm. Varying based on the seasonal water recharge, Urmia Lake extends 140 km towards north–south directions and 16 to 63 km in east–west direction. Urmia Lake is a part of the Azerbaijan plateau as well as the northwestern part of the Iranian plateau which are dominated by a diversity of alluvial salts derived from Cretaceous (e.g., grey limestone, sandstone, marl, and shale), basic igneous rocks, and Quaternary rocks^23^. Following cut off Tethys seaway from the ocean in the Late Cretaceous, the shallow intermountain lakes and lagoons are formed in the Tertiary in the Iranian plateau. Therefore, Urmia Lake likely is the remnants of the post-Tetyan sea and is rich in various salts and sediments.

Urmia Lake is recharged by more than 20 permanent and seasonal rivers and streams, mainly flowing in from the western side (Fig. 1). Seven specific rivers are the main feeders of the basin, named locally as Baranduz Chai (BAR), Ghadar Chai (GHA), Mahabad Chai (MAH), Nazloo Chai (NAZ), Shahar Chai (SHA), Simineh Chai (SIM), and Zola Chai (ZOL). These rivers are also the primary transporters of metal contaminated sediment and dissolved heavy metals from agricultural, urban, and industrial discharge to Urmia Lake basin^24^ which has raised concerns regarding the widespread dissemination of heavy metals through the entire basin and deteriorating the human and ecological health of Urmia Lake.

### Sediment sampling, preparation, and analyses

Sediment sampling locations were selected as near headwater to discharge points of lower estuary of each river/stream flowing into the Urmia Lake during winter in January–February, 2021 and during summer in August and September 2021. Using the standard procedure^25^, a total of 34 composite sediment samples were collected from 0 to10 cm depth at 34 locations corresponding to 7 revisers. The sampling sites were included 6, 3, 3, 12, 3, 4, and 2 sites for BAR, GHA, MAH, NAZ, SHA, SIM, and ZOL, respectively. For each sampling location at each river, composite sample was acquired by mixing three sub-samples. Heavy elements were analyzed in three replicates. An analogous sediment sampling procedure was followed for the summer sediments as for the winter sediments (n = 34).

Sediment samples were air-dried at 20–25 °C in ambient laboratory conditions, passed through a 2-mm sieve and stored for further laboratory analyses. All samples were analyzed to determine the main characteristics of sediments and the heavy metals concentration (Table 1). Heavy metal concentrations in sediment samples were measured using acid digestion method. 2 g of each sample was placed in a beaker and treated with 15 mL of concentrated HCl and HNO_3_ with a ratio of 12:4 at 130° C for 5 h. Finally, 4 mL of concentrated HCIO_4_ was added to the solution. The sediment was filtered and washed using 0.1 M HNO_3_ solution and diluted to 50 mL with deionized water. Using a flame atomic absorption spectrophotometry (model AA-6300, Shimadzu, Japan), the zinc (Zn), copper (Cu), cadmium (Cd), lead (Pb), and nickel (Ni) concentrations were determined. The precision was inspected using reagent blanks, replicates, determination of limits of detection (LOD), and the parallel analysis of international reference materials^26^. To calculate the LOD, three times the standard deviations of the concentration of the relevant metal was considered over six spiked samples^12^. The LODs were 2, 1, 0.01, 1, and 1 mg kg^−1^ for Zn, Cu, Cd, Pb, and Ni, respectively. Moreover, the standard solution concentrations were 0.5, 1, 1.5, 2, and 2.5 or 4, 6, 8, 10, and 12 mg l^−1^ for Zn and Cu; and 0.5, 1,1.5, 2 and 2.5 mg l^−1^ for Cd, Pb, and Ni.

**Table 1:** Analytical methods used to identify soil variables.

Soil variable	Protocol	References
Particle size distribution	Hydrometer method	^27^
pH	Soil-solution ratio of 1:5	^28^
EC	Soil-solution ratio of 1:5	^29^
OM	Potassium dichromate oxidation	^30^
CCE	Acid neutralization	^31^
ACCE	Neutral ammonium oxalate	^31^
Zn	Concentrated nitric acid	^32^
Cu	Concentrated nitric acid	^32^
Cd	Concentrated nitric acid	^33^
Pb	Concentrated nitric acid	^33^
Ni	Concentrated nitric acid	^33^

*EC* Electrical conductivity; *OM* Organic matter; *CCE* Calcium carbonate equivalent; *ACC* Active calcium carbonate.

Using an X-ray fluorescence (XRF) spectrometry (PANalytical, Netherlands), elemental analyses of sediment samples were estimated^34^. For each sediment sample, 15 g of sediment material was weighed, dried at 60 °C for 24 h, and then three drops of Triethanolamine (C_6_H_15_NO_3_) were added. After grinding the samples, 8 g of each was made into pellets and major element oxides were generated. The geochemical weathering indices were calculated using the following Eq. ^35^.

1 $$CIA = \frac{{Al_{2} O_{3} }}{{Al_{2} O_{3} + CaO + K_{2} O + Na_{2} O}} \times 100$$

Here *CIA* is the geochemical index of alteration and Al_2_O_3_, CaO, K_2_O, and Na_2_O are input moles of oxide molecules. CIA has been widely used to indicate the intensity of silicate weathering in the sedimentary basins^35,36^ .

### Evaluation of sediment contamination indices

Three pollution indices were used to determine the degree of metal contamination in sediments of the Urmia Lake basin and to determine whether metal toxicity and the potential health risks endanger the life quality and the ecosystem of this region.

#### Pollution index and Pollution load index^37^

2 $$PI = \frac{{C_{m} }}{{C_{b} }}$$

3 $$PLI = (PI_{1} \times PI_{2} \times PI_{3} \times PI_{n} )^{1/n}$$

where *C*_*m*_ is the heavy metal content of sediment (mg kg^−1^), *C*_*b*_ is the reference concentration of sediment metals (mg kg^−1^), *n* is the number of metals, and *PI* and *PLI* are Pollution Index and Pollution Load Index. In this study, the geochemical background was estimated using the Median Absolute Deviation (MAD) method^38^, accounting for the mean earth crust values^39^. The pollution index was categorized into unpolluted (PI ≤ 1), slightly polluted (1 < PI ≤ 2), moderately polluted (2 < PI ≤ 3), and highly polluted (PI > 3) levels^37^. Moreover, the PLI value was classified as no pollution (PLI < 1), moderate pollution (1 < PLI < 2), high pollution (2 < PLI < 3), and extremely high pollution (PLI > 3)^37^. Pollution and pollution load indices are effective tools to evaluate the magnitude of contaminants in the environment and are commonly used to appraise the status of heavy metals in various ecosystems^2,15,37^.

#### Assessment of potential ecological risk

The ecological risk of heavy metals pollution for aquatic organisms was assessed based on the productivity response of aquatic ecosystem to the potential heavy metal pollution^40^.

4 $$ER = \sum E_{r}^{i}$$

5 $$E_{r}^{i} = T_{r}^{i} P_{f}^{i}$$

6 $$P_{f}^{i} = \frac{{C_{0}^{i} }}{{C_{n}^{i} }}$$

where *ER* is the total ecological risk factor of the metals, $E_{r}^{i}$ is the single ecological risk factor, and $T_{r}^{i}$ is the metal toxicity response factor, which is equal to 5 mg kg^−1^ for Cu, Pb, and Ni, 30 mg kg^−1^ for Cd, and 1 mg kg^−1^ for Zn. $P_{f}^{i}$ is the contamination factor. $C_{0}^{i}$ is the metal concentrations in the sediment, and $C_{n}^{i}$ is the reference values for heavy metals^41^. The ER, ecological risk factor was explored within four intensity classes; low risk (ER < 150), moderate risk (150 ≤ ER < 300), considerable risk (300 ≤ ER < 600), and high risk (ER > 600)^40^.

#### Human health risk indices

The potential non-carcinogenic and carcinogenic health risks of heavy metals to children and adults via ingestion, inhalation, dermal contact was assessed separately using the following Eqs. ^18,42^:

7 $$ADD_{ing} = C \times \frac{{IR_{ing} \times EF \times ED}}{BW \times AT} \times 10^{ - 6}$$

8 $$ADD_{inh} = C \times \frac{{IR_{inh} \times EF \times ED}}{PEF \times BW \times AT}$$

9 $$ADD_{derm} = C \times \frac{SA \times AF \times ABS \times EF \times ED}{{BW \times AT}} \times 10^{ - 6}$$

Here *ADD*_*ing*_, *ADD*_*inh*_, and *ADD*_*derm*_ are the Average Daily Dose (ADD) uptake from sediment ingestion, inhalation, and dermal contact, respectively (mg element per kg^−1^ bodyweight day^−1^) and *C* is the heavy metal concentration in a given sediment sample (mg kg^−1^). Other parameters including *IR*_*ing*_*, AT, BW, IR*_*inh*_*, ED, EF, PEF, ABS, AF* and *SA* are presented in Table S1^42,43^.

Non-carcinogenic risk of heavy metals was calculated using the hazard quotient (HQ), represented by the quotient of the ADD of each heavy metal and the corresponding reference dose (Eqs. 10–11)^18,25,42^.

10 $$HI = \sum HQ_{I} = \sum \frac{{ADD_{i} }}{{RfD_{i} }}$$

11 $$THI = \sum HI$$

Here, HI and THI are hazard index and total hazard index, respectively. HI/THI > 1 represents a significant non-carcinogenic effect to the public, whereas HI/THI < 1 indicates no apparent non-cancer risk to the public.

Similarly, carcinogenic risk was calculated using Eqs. (12 and 13)^44^:

12 $$CR = ADD_{i} \times SF$$

13 $$TCR = \sum CR$$

Here, *CR* is the carcinogenic risk from each metal, *SF* is the slope factor (kg day mg^−1^), and *TCR* is the total carcinogenic risk indexes. The carcinogenic risk of heavy metals was categorized into negligible (CR/TCR < 1 × 10^−6^), admissible risk (1 × 10^−6^ < CR/TCR < 1 × 10^−4^), and impermissible risk (CR/TCR > 1 × 10^−4^) classes^44^.

The Toxicity Unit (TU) was obtained by determining the half maximal inhibitory concentration (IC_50_) of heavy metals extracted from the sediment material. The TU of chemicals in the mixture was calculated using the following Eqs. ^45^.

14 $$TU_{i} = \frac{{C_{i} }}{{IC_{50i} }}$$

15 $$\mathop \sum \limits_{i = 1}^{n} TU_{i} = \left( {\frac{{C_{1} }}{{IC_{{50_{1} }} }} + \frac{{C_{2} }}{{IC_{{50_{2} }} }} + \cdots \frac{{C_{n} }}{{IC_{{50_{n} }} }}} \right)$$

where TU_i_ is the toxic unit of ith heavy metal in the mixture, C_i_ is the concentration of ith heavy metal in the mixture at IC_50_ which is the median inhibition concentration.

### Data analysis

All descriptive statistics (e.g., maximum, mean, minimum, standard deviation, correlations, etc.) were conducted by Microsoft Excel 2016. The Analysis of Variance (ANOVA) was conducted in SAS (version 9.4)^46^ using GLM procedure. Least squared means were separated using Fisher’s least significant difference (LSD) test at 95% confidence interval. The normality of data distribution was satisfied according to Shapiro–Wilk > 0.9, otherwise log-transformation was performed. Principal component analysis (PCA) was performed to reduce the multicollinearity among variables and to identify the possible type of heavy metal pollution in the rivers.

## Results and discussion

### Basic characteristics of river sediments

A considerable variation was found in the distribution of clay (81 to 48.4 g kg^−1^), silt (145 to 656 g kg^−1^), and sand (38 to 821 g kg^−1^) particles among sediment materials. The associated coefficient of variations (CV) was 57, 59.5, and 41%, respectively. Statistical data related to the physicochemical properties of sediments and their main elements are reported in Table 2. The variations in particle size distribution located sediment material in seven textural classes ranging from loamy sand to silty clay. The high variability in particle size distribution suggests that different sets of geogenic and anthropogenic processes are enacted in the development and distribution of sediments in the rivers. The pH and CCE ranged from 7.4 to 8.2 and 31 to 251 g kg^−1^, respectively, indicating the dominancy of alkaline-calcareous condition. None of the sediment samples exhibited salinity conditions (EC > 4 dS m^−1^) with EC in the range of 0.3 to 1.4 dS m^−1^. A relatively low range of OM was found in all samples ranging from 7 to 61 g kg^−1^ with a mean value of 19 g kg^−1^. This range of OM coincides with the corresponding values in regional soils^47^. Except for pH, other sediments properties demonstrated above 35% of CV illustrating a wide range of variability in sediments’ physicochemical properties across the study rivers.

**Table 2:** Summary statistics of sediment properties.

Sediment variable	Max	Min	Mean	SD	CV (%)
Clay (g kg^−1^)	484	81	179	10.2	57.0
Silt (g kg^−1^)	656	145	412	24.5	59.5
Sand (g kg^−1^)	821	38	409	16.8	41.1
pH	8.2	7.4	7.7	0.31	4.0
EC (dS m^−1^)	1.4	0.3	0.5	0.35	70.0
OM (g kg^−1^)	61	7	19	1.2	63.2
CCE (g kg^−1^)	251	31	162	6.3	38.9
ACC (g kg^−1^)	43	7	25	1.3	52.0
SiO_2_ (%)	55.2	37.5	44.9	3.92	8.3
Al_2_O_3_ (%)	15.9	8.9	12.6	1.92	15.2
Fe_2_O_3_ (%)	10.0	4.8	6.7	1.41	21.0
CaO (%)	14.3	5.0	10.6	2.35	22.2
MgO (%)	17.2	2.4	5.5	3.43	62.4
K_2_O (%)	3.1	1.2	2.3	0.49	21.3
SO_3_ (g kg^−1^)	4.8	0.01	0.89	0.09	101.1
Na_2_O (%)	2.7	0.68	1.5	0.44	29.3
CIA	85.7	64.9	72.9	4.95	100

*OC* organic matter; *CCE* calcium carbonate equivalent; *ACC* active calcium carbonate; *CEC* cation exchange capacity; *EC* electrical conductivity; *SAR* sodium adsorption ratio.

The highest concentration among major elements was observed in SiO_2_, varying between 37.5 and 55.2%, with a mean percentage of 44.9%. This element followed in magnitude by Al_2_O_3_ (8.9–15.9%), CaO (5–14.3%), Fe_2_O_3_ (4.8–10%), MgO (2.4–17.2%), K_2_O (1.2–3.1%), Na_2_O (0.68–2.7%), SO_3_ (0.01–4.8% g kg^−1^) (Table 2). Considering the semi-arid climatic condition of the study region, higher levels of SiO_2_ and lower levels of Al_2_O_3_ may indicate that the silicate minerals forming the sediments of the area have not been subjected to severe weathering processes. Likewise, the Na_2_/K_2_O ratio was greater than 1 in the majority of sediment samples, implying an enrichment of potassium feldspar and the relatively intense weathering of Na-bearing minerals in the region^48,49^. The CIA value was in the range of 64.9 to 85.7% with a mean percentage of 72.9%, representing a moderate chemical weathering intensity of lithological materials (65% < CIA < 85%) in the region^36^.

### Heavy metals concentration in sediments

**Figure 2:**
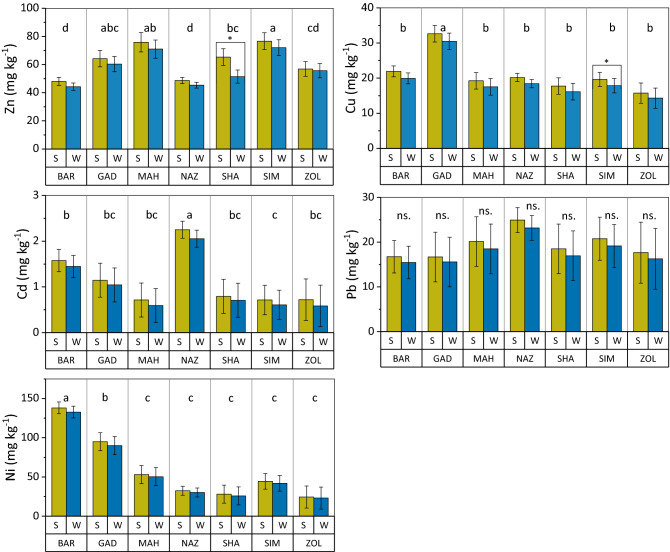
The comparison of the mean concentration of Zn, Cu, Cd, Pb, and Ni elements in the study rivers’ sediments during summer and winter. Different letters show significant differences in metal content among rivers pooled over seasons at P < 0.05 confidence level. ns. represents the lack of significat difference.

Heavy metal concentrations except for a few cases shown in Fig. 2, did not vary significantly between summer and winter periods, although an overall greater concentration during summer period was evident across all rivers. In summer, sediments contained 6.4–20.2%, 7.1–10%, 9.1–22.6%, 7.2–9.9%, and 5–8.4% higher Zn, Cu, Cd, Pb, and Ni contents than winter. Greater heavy metal concentrations during summer are likely due to the seasonal variations in rivers’ water fluxes as such water recharge to the river is limited in summer, generating less mobility and greater accumulation of heavy metals in the summer. Nevertheless, contradictory observations exist in the literature^50^. The positive relationship between heavy metals concentration in sediments and the river flow depth and rate has been thoroughly observed in previous studies^51,52^. The mean annual concentrations of heavy metals except Pb, varied significantly across studied rivers (Fig. 2). The greatest mean annual Zn, Cu, Cd, and Ni concentrations were observed in Sim, Gad, Naz, and Bar rivers, respectively. The observations indicate the diversity of contaminant resource surrounding the basin rivers. Descriptive statistics of the five heavy elements concentrations in the sediments of the Urmia Lake basin rivers are presented in Table 3. The mean values of heavy metals in the sediments were in the descending order of Ni > Zn > Pb > Cu > Cd which varied largely among the sampling points. The level of Zn, Cu, Cd, Pb, and Ni varied in the ranges of 32.6–87.5, 14.2–33.3, 0.42–4.8, 14.5–69.5, and 20.1–183.5 mg kg^-1^, respectively, for winter, and 35.3–92.5, 15.6–35.1, 0.47–5.1, 15.5–73.1, 23.2–188.3 mg kg^−1^ for summer. The obtained ranges are comparable with data found in previous studies in Asia^4,54–54^.

**Table 3:** Summary statistics of heavy metal in the study sediment during summer and winter.

Heavy metal	Season	Max	Min	Mean	SD	CV (%)
Zn	Winter	87.5	32.6	53.4A	12.9	24.2
	Summer	92.5	35.3	58.2a	14.1	24.3
Cu	Winter	33.3	14.2	19.2B	5.2	27.1
	Summer	35.1	15.6	21.3b	5.5	25.8
Cd	Winter	4.8	0.42	1.3C	0.83	63.8
	Summer	5.1	0.47	1.5b	0.88	55.0
Pb	Winter	69.5	14.5	19.1B	9.1	47.6
	Summer	73.1	15.5	20.61b	9.5	43.4
Ni	Winter	183.5	20.1	58.9A	45.3	76.9
	Summer	188.3	23.2	62.09a	46.7	72.4

The capital letters separate means of winter sediments’ heavy metal concentrations and lower-case letters separate the summer sediments’ mean values according to Fisher’s least significant difference (LSD) test at 95% confidence interval.

The variability of heavy metal concentrations, characterized by CV ranged from 24 to 77% (Table 3). Given the values of CV, heavy metals were grouped into two levels of moderate (Zn and Cu with 16% < CV < 36%) and high variability (Cd, Pb, and Ni with CV > 36%)^55^. These results highlight that the heavy metal variabilities were in a moderate to high level in the river sediments of the Urmia Lake basin which is likely associated with the spatial distribution of heavy metal discharge units along the rivers.

### Sediment properties affecting heavy metal adsorption

**Figure 3:**
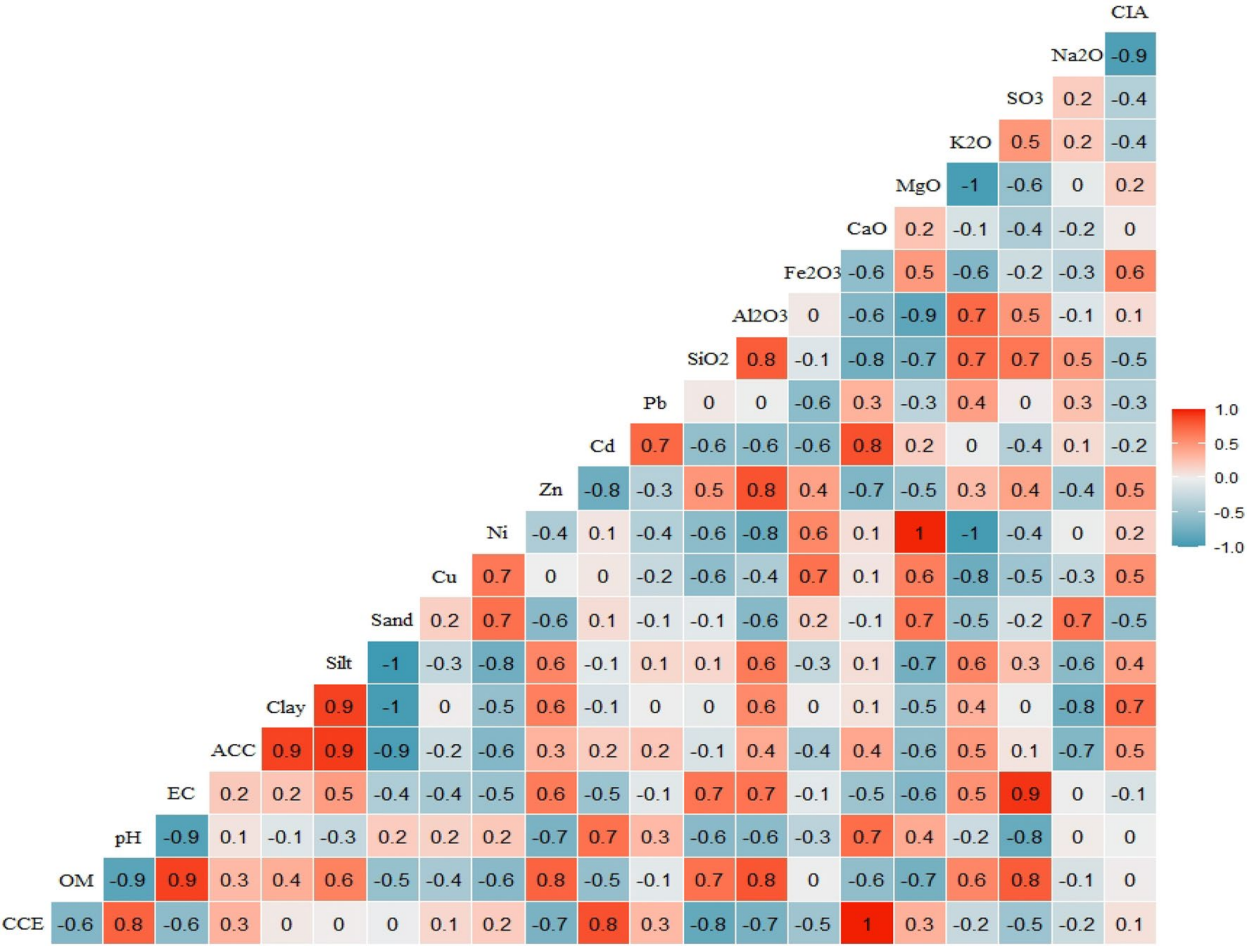
Pearson correlation coefficients among selected sediment attributes.

The Pearson's correlation analysis was conducted to identify the sediment properties that may exert a control on sediment’s heavy metal adsorption capacity (Fig. 3). The majority of heavy metals showed strong to moderate correlations with Al_2_O_3_ (r = 0.4 to 0.8) and Fe_2_O_3_ (r = 0.4 to 0.7) content, illustrating that Al and Fe oxides considerably increase the heavy metal retention capacity of sediments. These correlations are consistent with previous observations^11,56^, which highlighted the substantial increase of heavy metals absorbance in presence of aluminum and iron oxides in mineral particles. Significant positive correlations were found between Cd and Pb (p < 0.01) concentrations in given riverine sediment material whereas less significant correlation coefficient found between Ni and Cu, implying an analogy in transport-accumulation mechanism and sources of these metal pairs^57^. Similar observations are reported by Ustaoğlu and Islam^58^ in sediments of a few rivers in Giresun, northeast Turkey, and Shu et al.^4^ in the surface sediments of tidal flats along the north Jiangsu coast, China. The lack of significant correlation between Zn on one hand and Cu, Cd, Pb, and Ni on the other hand corroborate that this element may not have a common sources and transport mechanism with other metals.

### Sediment pollution indices

**Figure 4:**
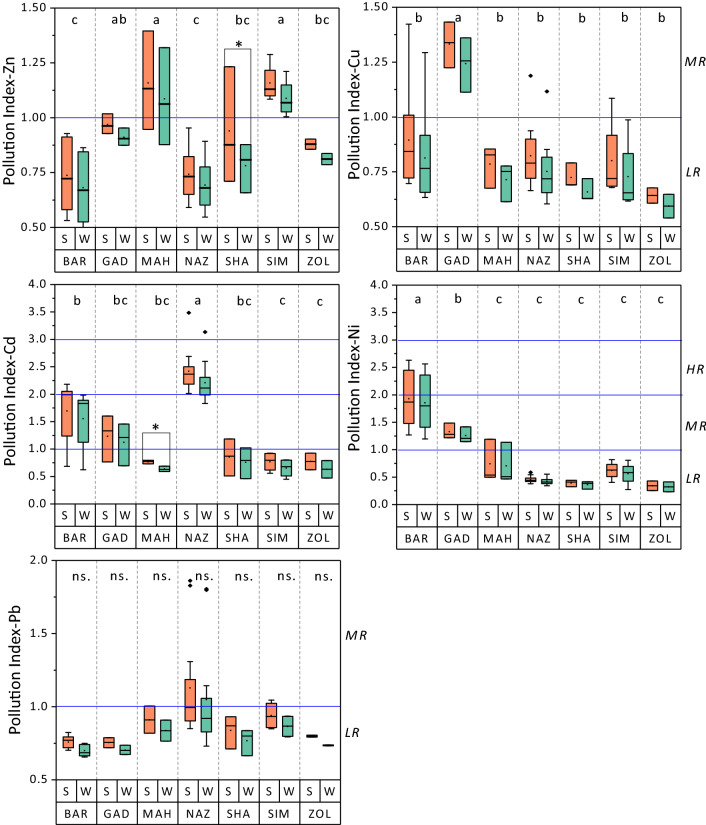
The comparison of the mean pollution index of Zn, Cu, Cd , Pb , and Ni between summer and winter in the study rivers’ sediments. Different letters represent significant differences in enrichment factor among rivers pooled over seasons at P < 0.05 confidence level. * represents significant differences between summer and winter measrements at P < 0.05. Horizontal lines demosnstarte the critical levels. LR is “Low Risk”, MR is “Midium Risk”, and HR is “High Risk”. ns. represents the lack of significat difference.

**Figure 5:**
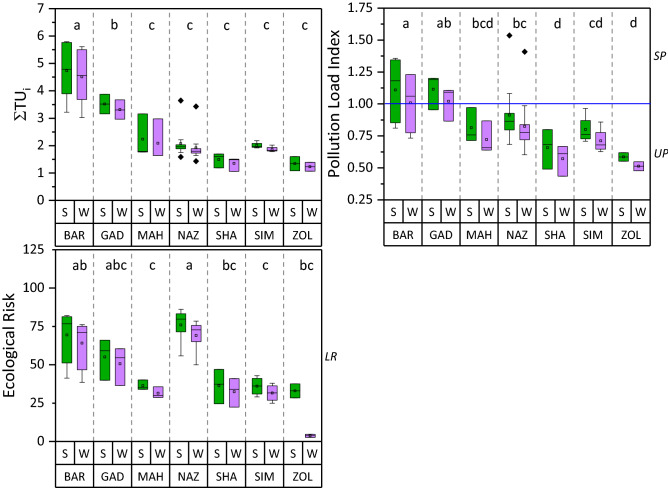
The comparison of the mean pollution load index (PLI), ecological risk (ER), and potential acute toxicity of heavy metals (∑TU) of selected heavy metals during summer and winter in the study rivers’ sediments. Different letters show significant differences in PLI, ER, and ∑TU among rivers pooled over seasons at P < 0.05 confidence level. Horizontal lines demosnstarte the critical levels. UP is “Unpolluted”, SP is “Slightly Polluted”, and LR is “Low Risk”.

The summary statistics for PI, PLI, and ER pollution indices are reported in Table 4. The PI values for sediment-associated heavy metals ranged between 0.53–1.4, 0.64–1.43, 0.51–5.48, 0.7–3.31, and 0.32–2.63 for Zn, Cu, Cd, Pb, and Ni in the summer and 0.49–1.32, 0.58–1.36, 0.45–5.13, 0.66–3.14, and 0.28–2.56 in the winter, respectively. These ranges of PI fall within unpolluted (PI ≤ 1) to slightly polluted (1 < PI ≤ 2) categories for Zn, Cu, and Pb, and slightly polluted to moderately polluted (2 < PI ≤ 3) categories in the mainstream of sediment samples according to Muller^59^. The ANOVA revealed significant differences in PI values among the rivers (Fig. 4). MAH and SIM rivers exhibited significantly greater levels of PI-Zn than BAR and NAZ rivers. Likewise, the GAD river had significantly the greatest PI-Cu among all rivers, even though the PIs for both Zn and Cu remained below the high-risk level (PI < 2). The PI-Pb did not exceed the low-risk level for almost all rivers, except the NAZ river which showed moderate-risk of PI, especially during the summer period. The greatest values of pollution index occurred regarding the Cd and Ni metals where PI-Cd and PI-Ni reached the high-risk level in NAZ and BAR rivers, respectively (Fig. 4). The mean comparisons convey no significant differences in PI during summer and winter periods for most rivers, even though an increase from 6.2–8.2% for Zn, 5.3–10.8% for Cu, 6.8–13.3% for Cd, 5.4–6.1% for Pb, and 2.7–14.3% for Ni in the summer versus winter period was evident (Fig. 5), indicating that the change of seasons can affect the concentration of heavy metals in sediments. It is allied with the greater summer temperature which accelerates the biodegradation of organic matter and releases binding metals^58,60^.

**Table 4:** The level and categorization of PI, PLI, Ei, and ER of the analyzed heavy metals during summer and winter.

Parameter	Pollution Index (PI)										
	Summer						Winter				
		Zn	Cu	Cd	Pb	Ni	Zn	Cu	Cd	Pb	Ni
Min		0.53	0.64	0.51	0.70	0.32	0.49	0.58	0.45	0.66	0.28
Max		1.40	1.43	5.48	3.31	2.63	1.32	1.36	5.13	3.14	2.56
Mean		0.88ab	0.86ab	1.59a	0.93ab	0.87b	0.80ns	0.79ns	1.44ns	0.8ns	0.82ns
SD		0.21	0.22	0.95	0.43	0.65	0.19	0.21	0.78	0.30	0.64
%Class of total samples	UP	74.5	79.4	38.2	79.5	67.7	82.3	85.3	41.2	97.1	67.7
	SP	23.5	20.6	23.5	17.6	23.5	17.6	14.7	41.7	–	23.5
	MP	–	–	35.4	–	8.8	–	–	14.7	–	8.8
	HP	–	–	2.9	2.9	–	–	–	2.9	2.9	–

	**Pollution Load Index (PLI)**										
		Summer					Winter				
		Min	Max	Mean	PLI < 1	1 < PLI < 2	Min	Max	Mean	PLI < 1	1 < PLI < 2
PLI		0.5	1.54	0.91	73.5%	26.5%	0.43	1.41	0.82	79.4	20.6

Parameter	Single Ecological Risk Factor (Ei)										
		Summer					Winter				
		Zn	Cu	Cd	Pb	Ni	Zn	Cu	Cd	Pb	Ni
Min		0.53	3.19	15.26	3.51	1.62	0.49	2.90	13.55	3.28	1.41
Max		1.40	7.16	164.52	16.54	13.15	1.32	6.80	154.03	15.72	12.81
Mean		0.88c	4.34b	50.07a	4.96b	4.51b	0.81C	3.97B	45.34A	4.60B	4.28B
SD		0.21	1.12	28.42	2.15	3.26	0.19	1.06	26.82	2.05	3.16
%Class of total samples	LR	100	100	44.2	100	100	100	100	47.1	100	100
	MR			52.9					52.9		
	CR			2.9							

	**Total Ecological Risk (ER)**										
		Summer					Winter				
		Min	Max	Mean	SD	%Class of total samples	Min	Max	Mean	SD	%Class of total samples
ER		24.6	189	64.4	21.2	LR = 97.1	21.6	177.1	59	20.1	LR = 97.1
						MR = 2.9					MR = 2.9

PI; UP = Unpolluted (PI < 1), LP = low pollution (1 < PI ≤ 2), MP = Moderate pollution (2 < PI ≤ 3), HP = High pollution (PI > 3).

Ei; LR = Low risk (Ei ≤ 40), MR = Moderate risk (40 < Ei ≤ 80), CR = Considerable risk (80 < Ei ≤ 160).

ER; LR = Low Risk (ER < 150), MR = Moderate risk (150 < ER < 300).

The capital letters separate means of winter sediments’ heavy metal concentrations and lower-case letters separate the summer sediments’ mean values according to Fisher’s least significant difference (LSD) test at 95% confidence interval.

*ns* represents the lack of significat difference.

The ANOVA revealed a significant difference among rivers in the PLI index (Fig. 5), even though the values did not exceed the range of the slightly polluted category (PLI < 1). The magnitude of PLI values among studied rivers were in the order of BAD > GAD > NAZ > MAH > SIM > SHA > ZOL (Fig. 5). PLI varied from 0.5 to 1.54 and 0.43 to 1.41 with a mean value of 0.91 and 0.82 in the summer and winter sediments, respectively, reflecting the range of unpolluted (PLI < 1) to the lower limits of slightly polluted (1 < PLI < 2) (Table 4). Nearly 73.5% and 26.5% of the summer sediment and 79.4% and 20.6% of the winter sediment showed PLI-no pollution and PLI- moderate pollution, respectively. The mean value of PLI was greater than 1 for BAR and GAD rivers in both summer and winter sediment samples, reflecting slight pollution in the sediments of these two rivers while the sediments of other rivers were nonpolluted.

The mean E_i_ varied significantly among heavy metals. The E_i_ values for the five heavy metals followed the descending order of Cd > Pb > Ni > Cu > Zn for both summer and winter sediments (Table 4). All metals exhibited a low ecological risk to the environment with E_i_ values lower than 40 except for Cd. The E_i_ values for Cd exceeded 80 in more than 52% of the total samples of both seasons, indicating Cd had considerable ecological risks in most sediment samples. Our results on E_i_-Cd are comparable to the previous studies where studied rivers were extending along vast urban or agricultural area^52,61^. It can be attributed to the fact that cadmium concentration in all rivers was way beyond the reference level of 0.3 mg kg^−1^ found naturally in shale, indicating a strong anthropogenic intervention, particularly in NAZ and BAR rivers which flow along larger farmland areas^62,63^. The influx of phosphate fertilizers applied to cropland, dumping of oily materials from water vehicles, and leakage of waste leachate from manufacturing units along the rivers are among the common reasons for high Cd concentrations^52,64^.

The ER values throughout the study region were in the range of 24.6–189 with a mean value of 64.4 during summer and 21.6–177.1 with a mean value of 59 during winter (Table 4), suggesting low to moderate ecological risk in 97.1% and 2.9% of both summer and winter sediments, respectively. The mean ER values were significantly different across the sediments of seven rivers and showed the descending order of NAZ > BAR > GAD > MAH > SHA > SIM > ZOL (Fig. 5). Similar to PLI, the mean ER values of the summer sediments were higher than the winter period, ranging from 8.2 to 17.8% (Fig. 5). The magnitude of ER values across rivers are comparable with their corresponding sediment metal concentration during both seasons.

The toxic unit (TU) was calculated to assess the impact of sediment pollutants on the aquatic organisms^45^. The values of TU decreased in the order of Ni > Cd > Pb > Zn > Cu during the summer and winter periods (Table 5), which aligns well with the previous studies^52^. The sum of individual TU values across heavy metals was less than 4 in 85 and 88% of sediment samples during summer and winter, respectively, indicating a low toxicity level of heavy metals to benthic organisms for most samples^65^. BAR and GAD rivers deduced significantly greater values of ∑TU than other rivers (Fig. 5). For both summer and winter samples, the contributions of each heavy metal to ∑TU were highly variable, with the highest contribution from Ni (66.1–66.9%), followed by Cd (15.7–16.2%), Pb (8.77–8.86%), Zn (6.73–6.83%), and Cu (3.9–4%). These results surmise a considerable possibility of the adverse effect of Ni and to a lower extent Cd on freshwater organisms. The high contributions of Ni and Cd may be relevant to their low PEL values, as suggested by several authors^4,66^. Analogous to the other pollution indices, ∑TU was 5 to 12% higher in sediments collected during summer than winter periods, indicating that the potential acute toxicity of heavy metals was affected by seasonal changes in flow characteristics.

**Table 5:** The level of toxic unit, sum of toxic units, and modified Hazard Quotient of the heavy metals during summer and winter.

Heavy metal	Toxic unit (TU)				
	Season	Max	Min	Mean	SD
Zn	Summer	0.112	0.294	0.185cd	0.045
	Winter	0.104	0.278	0.171BCD	0.041
Cu	Summer	0.079	0.178	0.108d	0.028
	Winter	0.072	0.169	0.099D	0.026
Cd	Summer	0.134	1.445	0.420bc	0.250
	Winter	0.119	1.353	0.38B	0.236
Pb	Summer	0.170	0.801	0.240bcd	0.104
	Winter	0.159	0.761	0.223BCD	0.099
Ni	Summer	0.644	5.231	1.792a	1.296
	Winter	0.559	5.096	1.704A	1.257

∑TUs	Sum of toxic units (∑TU)				
	Season	Max	Min	Mean	SD
	Summer	1.186	5.793	2.706	1.308
	Winter	1.058	5.611	2.538	1.272

The capital letters separate means of winter sediments’ heavy metal concentrations and lower-case letters separate the summer sediments’ mean values according to Fisher’s least significant difference (LSD) test at 95% confidence interval.

### Human health risk assessment

#### Non-carcinogenic heavy metal risk

The health risk of Zn, Cu, Cd, Pb, Cd to induce non-carcinogenic diseases via ingestion, inhalation, and dermal contact was calculated for children and adults separately (Table 6). The HQ values of ingestion pathway was maximum at the range of 0.0001–0.267 for all metals and both age groups which followed by HQ_der_ (a range of 2.18E−08) and HQ_inh_ (a range of 4.35E−05–0.167). This result implies that the non-carcinogenic risk of diseases caused by the ingestion of all heavy metals from the riverine sediment of the region is much more hazardous than that by dermal contact and inhalation. These observations are analogous to the findings of the studies conducted by Proshad et al.^52^ and Şimşek et al.^2^. The HQ ranking of metals demonstrated the descending order of Pb > Ni > Cd > Cu > Zn for three exposure pathways and both age groups and seasons. In accordance with the previous studies^8,58^, the observed trend indicates a greater role of Pb in the incidence of non-carcinogenic risk than the other metals.

**Table 6:** The minimum, maximum, and mean values of HQ, HI, CR, and TCR of heavy metals from ingestion, inhalation, and dermal contact of sediments.

Heavy metal		HQ-Summer				HQ-Winter			
		Ingestion	Inhalation	Dermal	HI	Ingestion	Inhalation	Dermal	HI
**HQ and HI-Child**									
Zn	Min	0.0015	4.94E−08	0.0008	0.0024	0.0014	4.35E−08	0.0008	0.0022
	Max	0.0039	1.30E−07	0.0022	0.0062	0.0037	1.17E−07	0.0021	0.0058
	Mean	0.0025	8.14E−08	0.0014	0.0039	0.0023	7.17E−08	0.0013	0.0036
Cu	Min	0.0050	1.64E−07	0.0028	0.0078	0.0045	1.42E−07	0.0026	0.0071
	Max	0.0112	3.69E−07	0.0063	0.0176	0.0107	3.33E−07	0.0060	0.0167
	Mean	0.0068	2.23E−07	0.0038	0.0106	0.0062	1.94E−07	0.0035	0.0097
Cd	Min	0.0121	3.97E−07	0.0068	0.0189	0.0108	3.36E−07	0.0060	0.0168
	Max	0.1306	4.28E−06	0.0734	0.2040	0.1222	3.82E−06	0.0688	0.1910
	Mean	0.0397	1.30E−06	0.0224	0.0621	0.0360	1.12E−06	0.0202	0.0562
Pb	Min	0.0567	1.86E−06	0.0010	0.0878	0.0530	1.66E−06	0.0348	0.0189
	Max	0.2673	8.77E−06	0.0105	0.4208	0.2541	7.94E−06	0.1667	0.2040
	Mean	0.0802	2.63E−06	0.0032	0.1232	0.0744	2.32E−06	0.0488	0.0621
Ni	Min	0.0148	4.87E−07	0.0084	0.0232	0.0129	4.02E−07	0.0041	0.0170
	Max	0.1205	3.95E−06	0.0678	0.1883	0.1174	3.67E−06	0.0376	0.1550
	Mean	0.0413	1.35E−06	0.0232	0.0645	0.0392	1.23E−06	0.0127	0.0520
THI					0.2643				0.2457
**HQ and HI-Adult**									
Zn	Min	0.00016	2.35E−08	4.70E−05	0.00021	0.0001	2.18E−08	4.35E−05	0.0002
	Max	0.00042	6.17E−08	1.23E−04	0.00055	0.0004	5.83E−08	1.17E−04	0.0005
	Mean	0.00027	3.88E−08	7.76E−05	0.00034	0.0002	3.59E−08	7.17E−05	0.0003
Cu	Min	0.0005	7.81E−08	1.56E−04	0.0007	0.0005	7.10E−08	1.42E−04	0.0006
	Max	0.0012	1.76E−07	3.51E−04	0.0016	0.0011	1.67E−07	3.33E−04	0.0015
	Mean	0.0007	1.06E−07	2.12E−04	0.0009	0.0007	9.72E−08	1.94E−04	0.0009
Cd	Min	0.0013	1.89E−07	3.78E−04	0.0017	0.0012	1.68E−07	3.36E−04	0.0015
	Max	0.0140	2.04E−06	4.08E−03	0.0181	0.0131	1.91E−06	3.82E−03	0.0169
	Mean	0.0043	6.21E−07	1.24E−03	0.0055	0.0039	5.62E−07	1.12E−03	0.0050
Pb	Min	0.0061	8.87E−07	1.77E−03	0.0078	0.0057	8.29E−07	1.66E−03	0.0073
	Max	0.0286	4.18E−06	8.35E−03	0.0370	0.0272	3.97E−06	7.94E−03	0.0351
	Mean	0.0086	1.25E−06	2.51E−03	0.0111	0.0080	1.16E−06	2.32E−03	0.0103
Ni	Min	0.0016	2.32E−07	4.64E−04	0.0021	0.0014	2.01E−07	4.02E−04	0.0018
	Max	0.0129	1.88E−06	3.77E−03	0.0167	0.0126	1.83E−06	3.67E−03	0.0162
	Mean	0.0044	6.45E−07	1.29E−03	0.0057	0.0042	6.13E−07	1.23E−03	0.0054
THI					0.0235				0.0219

	CR-Summer				CR-Winter				
	Ingestion	Inhalation	Dermal	TCR	Ingestion	Inhalation	Dermal	TCR	
**CR and TCR-Child**									
Cd	Min	9.08E−06	2.98E−09	5.11E−06	1.42E−05	8.06E−06	2.52E−09	4.54E−06	1.26E−05
	Max	9.79E−05	3.21E−08	5.51E−05	1.53E−04	9.17E−05	2.87E−08	5.16E−05	1.43E−04
	Mean	2.98E−05	9.78E−09	1.68E−05	4.66E−05	2.70E−05	8.43E−09	1.52E−05	4.22E−05
Pb	Min	1.69E−06	5.54E−11	9.50E−07	2.64E−06	1.54E−06	4.93E−11	8.87E−07	2.43E−06
	Max	7.95E−06	2.61E−10	4.47E−06	1.24E−05	7.38E−06	2.36E−10	4.25E−06	1.16E−05
	Mean	2.39E−06	7.83E−11	1.34E−06	3.73E−06	2.16E−06	6.91E−11	1.24E−06	3.40E−06
**CR and TCR-Adult**									
Cd	Min	9.72E−07	1.42E−09	2.84E−07	1.26E−06	8.63E−07	1.26E−09	2.52E−07	1.12E−06
	Max	1.05E−05	1.53E−08	3.06E−06	1.36E−05	9.81E−06	1.43E−08	2.87E−06	1.27E−05
	Mean	3.19E−06	4.66E−09	9.31E−07	4.13E−06	2.89E−06	4.22E−09	8.43E−07	3.74E−06
Pb	Min	1.81E−07	2.64E−11	5.28E−08	2.33E−07	1.69E−07	2.47E−11	4.93E−08	2.18E−07
	Max	8.51E−07	1.24E−10	2.49E−07	1.10E−06	8.09E−07	1.18E−10	2.36E−07	1.05E−06
	Mean	2.55E−07	3.73E−11	7.46E−08	3.30E−07	2.37E−07	3.46E−11	6.91E−08	3.06E−07

**Figure 6:**
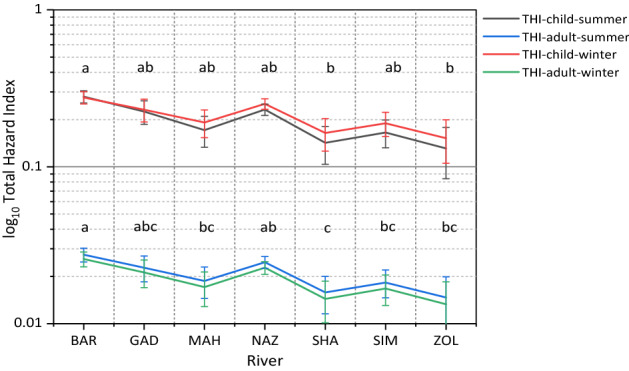
The comparison of the mean total hazard index of selected heavy metals between summer and winter for children and adult in the study rivers sediments. Different letters represent significant differences in Total Hazard Index among rivers pooled over seasons at P < 0.05 confidence level.

For both children and adults, the ranking of the HI values followed the order Pb > Ni > Cd > Cu > Zn during summer and winter―similar to the order observed for HQ values (Table 6). The findings confirmed that Pb and Zn had the maximum and minimum contribution to non-carcinogenic health risk, respectively. However, the HI values of all heavy metals as well as THI for children and adults were < 1, illustrating no significant non-carcinogenic risk in the analyzed sediments. In all sediment samples, children appeared to have significantly higher potential for non-carcinogenic health risk, HQ (via ingestion, inhalation, and dermal contact), HI (4.8 to 10.5 time), THI (10.2 to 10.3 time) compared with adult. The higher susceptibility of children to the health impacts of heavy metals is due to their behavioral characteristics―e.g., more outdoor activities and oral and finger practice^67^. Heavy metals can cause various health issues in children e.g., skeletal injuries, cardiovascular disease, or respiratory system and productivity^68^. For both summer and winter sediments, the mean values of THI across the seven rivers followed a descending order as BAR > NAZ > GAD > SHA > MAH > SIM > ZOL and BAR > NAZ > GAD > MAH > SIM > SHA > ZOL for children and adult, respectively (Fig. 6). The THI calculated based on summer sediments where higher than those of winter, ranging from 3 to 28% and 6.6 to 10.4% for child and adults, respectively.

#### Carcinogenic heavy metal risk

The carcinogenic risk potential (CR) of Cd and Pb was calculated for children and adult through ingestion (CR_ing_), inhalation (CR_inh_), and dermal contact (CR_der_) of sediments (Table 6). For both children and adults and during both seasons, the CR of different exposure pathways were in the order of CR_ing_ > CR_der_ > CR_inh_, a similar rank to those of non-carcinogenic risk (HQ and HI). During summer, the TCR (∑CR) values for Cd and Pb in children and adults ranged from 1.4E−05 to 1.53E−04 and 1.26E−06 to 1.36E−06 for Cd and from 2.64E−06 to 1.24E−05 and 2.33E−07 to 1.1E−06 for Pb, respectively. Regarding the winter, the corresponding ranges of TCR-Cd were 1.26E−05 to 1.43E−04 for children and 1.21E−06 to 1.27E−05 for adult while TCR-Pb ranged from 2.43E−06 to 1.16E−05 for children and 2.18E−07 to 1.05E−06 for adult. These results suggest that both children and adult were almost within tolerable risks (1 × 10^−6^ to 1 × 10^−4^) of carcinogenic hazards to human health. The mean value of TCR (Cd + Pb) was in rang of 4.56E−05 over winter to 5.03E−05 in summer and 4.05E−06 to 4.46E−06 for children and adult, respectively. The comparison of these data implies that the risk of TCR (Cd + Pb)-initiated cancer in children (5 case of cancer for every 1,00,000 inhabitants) was 11.2 to 11.3 times higher than in adult (4 to 5 case of for every 1,000,000 inhabitants). This distinction may also be explained by children’s behavioral patterns which encourages the propensity for their skin particularly hand-to-mouth activities^67^.

**Figure 7:**
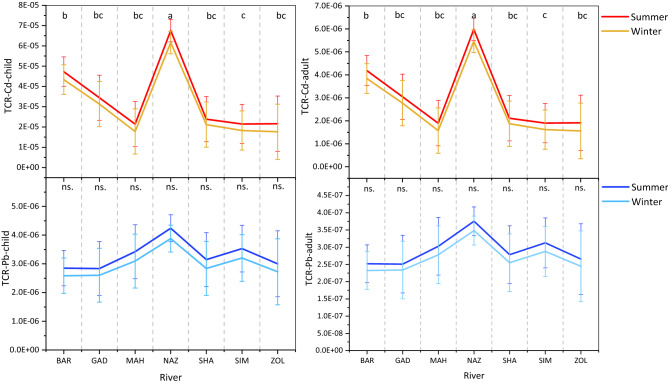
The comparison of the mean total carcinogenic risk (TCR) of Cd and Pb and for children and adult among the study rivers’ sediments. Different letters represent significant differences in enrichment factor among rivers pooled over seasons at P < 0.05 confidence level. ns. represents the lack of significat difference.

For both children and adults, the mean values of TCR across the sediments of seven rivers followed a descending order as NAZ > BAR > GAD > SHA > SIM > MAH > ZOL and NAZ > SIM > MAH > SHA > ZOL > BAR > GAD for Cd and Pb, respectively (Fig. 7). These results reflect a substantial variation in the spatial distribution of TCR as well as heavy metals concentration among riverine sediments of the region which is analogous to other studies in the literature^61,67,69^. Moreover, the mean values of TCR were significantly higher in the bulk of sediment samples during summer than those during winter (Fig. 7). The difference might be attributed to the slower flow rate during summer leading to heavy metal precipitation as well as intensified input from farmlands during summer^61^. Therefore, the sediment poses higher health risk during dry season, summer than the wet season, winter.

### Multivariate analysis

**Figure 8:**
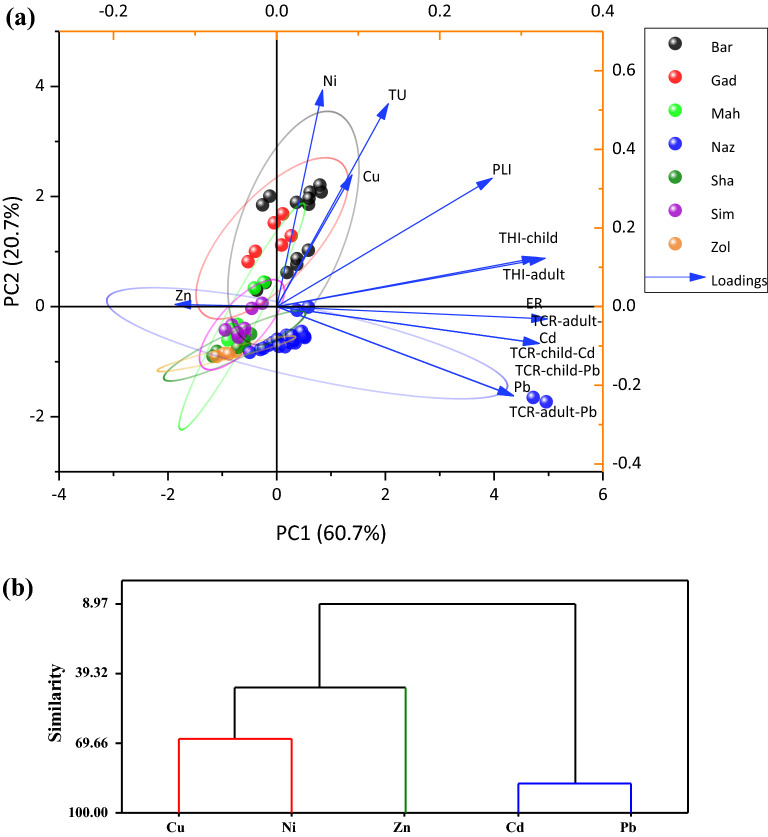
Biplot illustrates the PCA analysis (**a**) and dendrogram illustrates the hierarchical cluster analysis (**b**) of heavy metals in the sediment of the study rivers. Concentration ellipses around data points represent 95% confidence zone.

Principal component analysis for riverine sediments’ heavy metal concentrations and associated ecological and health risk indices has been provided in Fig. 8. The first and second PCs cumulatively explained 81.35% of the variability in the dataset. PC1 was associated with greater coefficients of ER, THI (children and adults), TCR-Cd (children and adults), Cd, TCR-Pb (children and adults), Pb, and PLI while PC2 was associated with greater coefficients of Ni, TU, Cu, and PLI. Pb and Cd exhibited high variability (CV > 36) and a significant positive correlation (r = 0.75, p < 0.01), indicating that Cd and Pb are most likely initiated from the same sources (e.g., discharge of urban wastewater, run-off from cropland or leachate from the waste dumping). Cd and Pb are considered two primary metals with industrial and urban origin (e.g., residues from industrial activities, traffic emission, and fuel combustion) and farm agrochemical (e.g., fertilizers and pesticides)^4,52,69,70^. Greater coefficients of variables within each PC indicates the collinearity among variables. The collinearity among heavy metals may indicate a mechanistic relationship between certain heavy metals that stimulate the co-occurrence of one another. Zn did not show collinearity with any of the other variables and was mainly explained by PC3 (Table 7), suggesting that Zn is likely to be mainly initiated from lithogenic origins such as natural weathering of Zn-bearing minerals or rocks as found in other studies^14,71–73^. In other studies, Zn is reportedly associated with basic minerals, particularly shales, the compounds that are predominant in the study region^11^. Regarding rivers, BAR and GAD showed a stronger sign of pollution with Ni and Cu metals and demonstrated a greater potential for acute toxicity, ∑TU. The lowest risk of acute toxicity was relevant to SIM, ZOL, and SHA rivers. Among all rivers, NAZ exhibited the greatest association with Pb and Pb-associated indices. The findings of the loading plot of the PCA and hierarchical cluster analysis (CA) (Fig. 8), which was performed to confirm the PCA data, showed the five heavy metals were categorized into three groups: Cd–Pb, Cu-Ni, and Zn. The findings of the CA were consistent with the PCA results. Overall, the data collected during the study are both representative and practically informative about the pollution and risk health of heavy metals in the river sediments of the Urmia Lake basin.

**Table 7:** Principal component analysis of heavy metals in the study sediment.

Heavy metal	PC1	PC2	PC3
Zn	–0.218	0.14	0.929
Cu	0.207	0.834	0.357
Cd	0.898	0.108	–0.327
Pb	0.926	–0.107	0.10
Ni	–0.238	0.81	–0.346
Eigenvalues	1.958	1.376	1.068
%Variance	39.19	27.52	21.35
Cumulative % variance	39.19	66.68	88.04

## Conclusion

We studied the distribution, source apportionment, contamination statuses, ecological impacts, and human health risk of Zn, Cu, Cd, Pb, and Ni in river sediments of the Urmia Lake basin, NW of Iran. The mean concentration of five heavy metals analyzed in the sediments of 7 rivers and during summer and winter was as follows: Ni > Zn > Pb > Cu > Cd with different spatial and temporal distributions along the rivers. Multivariate statistical methods show that the study sediments are under the influence of anthropogenic origin particularly by Cd and Pb. Approximately 73.5% of the summer sediment and 79.4% of the winter sediment showed “no pollution” (PLI < 1). Based on the ER index, 97.1% of both summer and winter sediments were ranked below “low ecological risk” and Cd is the primary heavy metal accountable for ecological threats followed by Pb, Ni, Cu, and Zn. The HI and CR for exposure of both children and adults to heavy metals fell within the accepted standard levels (HI < 1, 1 × 10^−6^ < CR < 1 × 10^−4^), signifying no non-carcinogenic and carcinogenic risk of the metals in the study region. The value of PI, PLI, ER, HQ, HI, and CR of sediments were higher in summer than those in winter, implying that the study sediments were under a higher intensity of heavy metals loads during summer. This study brings insight into the source, dissemination potential, and human health risk of heavy metals introduced from various resources to the river ecosystems in Urmia Lake basin. This information is expected to raise awareness about the facilitated introduction of heavy metals to the food chain through intensified dissemination through river flow. Continuous monitoring of heavy metals in water–sediment-aquatic biota system in Urmia Lake basin is essential for safeguarding the human, animal, and environmental health.

## Supplementary Information

Supplementary Information.

## Data Availability

The datasets used and/or analyzed during the current work are available from the corresponding author on reasonable request.
