# Supplementary material for: Distribution, source apportionment, and risk analysis of heavy metals in river sediments of the Urmia Lake basin
Source: Sci Rep. 2022 Oct 19;12:17455. doi: 10.1038/s41598-022-21752-w (PMC9582006; doi:10.1038/s41598-022-21752-w)
Supplement: Supplementary file 1 — Supplementary Information. [file 41598_2022_21752_MOESM1_ESM.docx]

**Distribution, source apportionment, and risk analysis of heavy metals in river sediments of the Urmia Lake basin**

Salar Rezapour ^1*^, Farrokh Asadzadeh ^2^, Amin Nouri ^3^, Habib Khodaverdiloo^4^, and Mohammad Heidari^5^

*^1, 2, 4^ Soil Science Department, Urmia University, P.O. Box 165, Urmia, 57134, I.R. Iran*

*^3^ Hermiston Agricultural Research and Extension Center, Oregon State University, Hermiston, OR 97838, USA*

*^5^Department of Epidemiology , School of Medicine,Urmia University of Medical Sciences, Urmia, Iran*

Salar Rezapour, Soil Science Department, Urmia University, P.O. Box 165, Urmia, 57134, I.R. Iran; E-mail: [S.Rezapour@urmia.ac.ir](mailto:S.Rezapour@urmia.ac.ir), and [S_Rezapour2000@yahoo.com](mailto:S_Rezapour2000@yahoo.com)

**Table S1** description and reference value of basic parameters for risk assessment models (USEPA, 2011; USEPA, 2014).

| **Parameter** | **Definition** | **Unit** | **Child** | **Adult** |
| --- | --- | --- | --- | --- |
| IR_ing_ | Ingestion rate | mg d^-1^ | 200 | 100 |
| EF | Exposure frequency | day year^-1^ | 350 | 350 |
| ED | Exposure duration | year | 6 | 30 |
| BW | Average body weight | kg | 15 | 70 |
| AT | Average life span for heavy metal | - | ED × 365 (2190) | ED × 365 (10950) |
| IR_inh_ | Inhalation rate | m^3^ d^-1^ | 8.5 | 16.1 |
| PEF | Particulate emission factor | m^3^ kg^-1^ | 1.32×10^9^ | 1.32×10^9^ |
| SA | Exposed skin area | cm^2^ | 2800 | 3950 |
| AF | Skin adhesive factor | mg cm^-2^ d^-1^ | 0.2 | 0.07 |
| ABS | Skin absorption factor | - | 1×10^-3^ | 1×10^-3^ |
